# Neuritin Attenuates Cognitive Function Impairments in Tg2576 Mouse Model of Alzheimer's Disease

**DOI:** 10.1371/journal.pone.0104121

**Published:** 2014-08-07

**Authors:** Yoori Choi, Kihwan Lee, Junghwa Ryu, Hyoun Geun Kim, A Young Jeong, Ran-Sook Woo, Jun-Ho Lee, Jin Won Hyun, Seokyung Hahn, Joung-Hun Kim, Hye-Sun Kim

**Affiliations:** 1 Department of Pharmacology and Biomedical Sciences, Neuroscience Research Institute, College of Medicine, Seoul National University, Seoul, Republic of Korea; 2 National Research Laboratory for Pain, Dental Research Institute and Department of Neurobiology and Physiology, School of Dentistry, Seoul National University, Seoul, Republic of Korea; 3 Department of Life Science, Pohang University of Science and Technology (POSTECH), Pohang, Republic of Korea; 4 Department of Anatomy and Neuroscience, School of Medicine, Eulji University, Daejeon, Republic of Korea; 5 Department of Emergency Medical Technology, Daejeon University, Daejeon, Republic of Korea; 6 Department of Biochemistry, School of Medicine, Jeju National University, Jeju, Republic of Korea; 7 Medical Research Collaborating Center, Seoul National University Hospital, Seoul, Republic of Korea; 8 Seoul National University Bundang Hospital, Seoul National University College of Medicine, Sungnam, Republic of Korea; University of Wurzburg, Germany

## Abstract

Neuritin, also known as CPG15, is a neurotrophic factor that was initially discovered in a screen to identify genes involved in activity-dependent synaptic plasticity. Neuritin plays multiple roles in the process of neural development and synaptic plasticity, although its binding receptor(s) and downstream signaling effectors remain unclear. In this study, we found that the cortical and hippocampal expression of neuritin is reduced in the brains of Alzheimer's disease (AD) patients and demonstrated that viral-mediated expression of neuritin in the dentate gyrus of 13-month-old Tg2576 mice, an AD animal model, attenuated a deficit in learning and memory as assessed by a Morris water maze test. We also found that neuritin restored the reduction in dendritic spine density and the maturity of individual spines in primary hippocampal neuron cultures prepared from Tg2576 mice. It was also shown that viral-mediated expression of neuritin in the dentate gyrus of 7-week-old Sprague-Dawley rats increased neurogenesis in the hippocampus. Taken together, our results demonstrate that neuritin restores the reduction in dendritic spine density and the maturity of individual spines in primary hippocampal neurons from Tg2576 neurons, and also attenuates cognitive function deficits in Tg2576 mouse model of AD, suggesting that neuritin possesses a therapeutic potential for AD.

## Introduction

Alzheimer's disease (AD) is the most common form of dementia and is characterized by two neuropathological hallmarks, neuritic plaques and neurofibrillary tangles. AD primarily targets synapses, and synaptic loss and dysfunction have been reported to be well correlated with cognitive dysfunction in AD [1]–[3]Immunoreactivity against synaptophysin, a presynaptic marker protein, is reduced by approximately 25% in the brains of patients with mild AD [4].

Neuritin, also known as candidate plasticity gene 15 (CPG 15), encodes a small, extracellular glycosylphosphatidylinositol (GPI)-anchored cell surface protein [5], [6] that was first identified in a screen for activity-regulated genes induced by kainate stimulated seizure in the rat dentate gyrus [7]. Neuritin is known as a critical regulator for dendritic outgrowth, maturation, and axonal regeneration [6], [8]–[11]. Its gene is located within the 6p2424-p25 interval on chromosome 6 [12]. 3 cyclic AMP responsive elements were identified in the promoter region of the gene, suggesting that neuritin expression is mediated by cyclic AMP responsive element binding protein (CREB). An *in vivo* study showed an important role for CREB in activity-dependent neuritin expression in the barrel cortex of control and CREB α, Δ-knockout mice. After eliciting receptive field plasticity by whisker trimming, the neuritin expression level increases in the barrel corresponding to the spared whisker. However, in CREB mutant mice, neuritin expression is not induced to the same extent as in wild-type (wt) littermates, indicating that CREB is necessary for neuritin regulation during receptive field plasticity in the adult cortex [13].

During developmental stages, neuritin expression has been shown to be continuously increased from embryonic day 12 to adulthood in the cortex and hippocampus [6]. In early embryonic development, neuritin is expressed in multiple brain regions and acts as a survival factor for neural progenitors and differentiated neurons [8], [14]. Later in development, neuritin promotes the growth and stabilization of axonal and dendritic arbors with synapse formation and maturation [8], [10], [15].

Here we demonstrate that the expression of neuritin was significantly decreased in the hippocampus and cerebral cortex of AD patients compared to age-matched control subjects. When transiently expressed in primary cortical neuron cultures, neuritin increased the level of synaptophysin, a presynaptic marker, which represents an increase in synaptogenesis. Importantly, lentiviral-mediated expression of neuritin in the dentate gyrus of Tg2576 mice harboring the Swedish double mutated human APP695 gene attenuated the deficits in learning and memory. In addition, neuritin rescued the reduction in the dendritic spine density and the numbers of mushroom-typed mature spines in primary hippocampal neuron cultures from Tg2576 mice. We also found that BrdU/NeuN staining increased in the hippocampal region of the Sprague-Dawley (SD) rats expressing neuritin. Collectively, neuritin possesses a therapeutic potential for neurodegenerative diseases such as AD by upregulating synaptogenesis and neurogenesis in brains.

## Materials and Methods

### Reagents

Antibodies were purchased as follows: anti-NeuN (Millipore, MA, USA), anti-BrdU (Abcam, Cambridge, UK), anti-neuritin (R&D systems, MI, USA), anti- beta-actin (anti-β- actin, Santa Cruz, CA, USA), anti-synaptophysin (Millipore, MA, USA), anti-glial fibrillary acidic protein (anti-GFAP, Dakocytomation, Glostrup, Denmark) antibodies. Neuritin peptide was purchased from Abcam.

### Human AD brains

Frozen and paraffin-embedded tissues of from 69 to 98 years old-AD and age-matched control subjects were obtained from the Netherlands Brain Bank (http://www.brainbank.nl/about-us/the-nbb/). AD tissues were diagnosed with neuropathological evidence using the criteria for Braak & Braak stage V or VI. The neuropathological diagnosis for non-demented controls was the neuropathological criteria for Braak & Braak stage 0 or I. To measure the levels of neuritin mRNA in the cortex and hippocampus, brain tissues from 3 age-matched control subjects (69, 79 and 80 years old) and 4 AD patients (67, 75, 81 and 87 years old) were used. To measure the levels of neuritin in the hippocampus, paraffin-embedded sections from 3 age-matched control subjects (69, 78 and 98 years old) and 3 AD patients (69, 75, and 78 years old) were used. All experiments were performed according to guidelines that were approved by the Experiments of Ethics Committee of Seoul National University.

### Experimental animals

All of the animal experimental procedures were approved by the Animal Care Committee of Seoul National University (Approval number: SNUIBC-080919-1). Experimental animals were given *ad libitum* access to food and water. Tg2576 mice harboring the Swedish double mutated human APP695 gene were obtained from Taconic (German Town, NY, USA). The production, genotyping, and background strain (C57BL/6_SJL) of Tg2576 mice used in this study were described previously [16]. For the Morris water maze test, male experimental animals were used in each group as follows: wt-EGFP, wt-neuritin, Tg2576-EGFP, Tg2576-neuritin. For the evaluation of neurogenesis as assessed with GFP/NeuN/BrdU staining, 7-week-old SD (Koatech, Seoul, Korea) rats weighing 200–250 g were used. All the animals were group-housed, except for the Tg2576 mice.

### Lentiviral-mediated expression of neuritin

PCR-amplified neuritin cDNA was subcloned into an IRES-EGFP lentiviral vector. The viral particles were prepared and concentrated by Macrogen (Seoul, South Korea). The viral particles from IRES-EGFP or neuritin were bilaterally injected into the dentate gyrus of the hippocampus of 13-month-old wt and Tg2576 mice 2.1 mm posterior to the bregma (AP), 1.8 mm lateral to the midline (ML) and 1.2 mm ventral to the surface of the skull (DV) or unilaterally injected into the dentate gyrus of the hippocampus of 7-week-old SD rats 3.5 mm posterior to the bregma (AP), 2.0 mm lateral to the midline (ML) and 4.0 mm ventral to the surface of the skull (DV), according to the Brain Atlas.

### Morris water maze test

The water maze apparatus, which is a circular pool (140 cm in diameter, 45 cm high), was filled with a 21–23°C water/dry milk powder mixture to create an opaque setting and located in a laboratory that contained prominent extra-maze cues. The animals are required to find a submerged platform in the pool using the spatial cues. The paradigm consisted of 3 trials per day over 5 consecutive days in which the mice were placed at one of 3 different start positions for each trial and given 60 seconds to locate the hidden platform. The latency time to escape onto the hidden platform was recorded. 24 h after the last day of spatial training, the probe test was carried out by removing the platform and allowing each mouse to swim freely for 60 s. The amount of time that each mouse spent swimming in each quadrant was recorded. Data collection was automated by a video image motion analyzer (Ethovision, Noldus Information Technology h.v., Netherlands).

### Quantitative real-time RT-PCR (qRT-PCR)

Total RNA was extracted from tissue using an RNA purification kit (Qiagen, Hilden, Germany) and 0.5 µg of RNA was processed for cDNA synthesis using oligo (dT)_20_ primers and SuperScript III reverse transcriptase (Invitrogen, CA, USA). The cDNA was then amplified using a 7500 Fast Real-Time PCR system (Applied Biosystems, CA, USA), employing the ΔΔCt method with SYBR Green I (Roche, CA, USA). The primers used are as follows: neuritin forward: 5′-CGAGA GAGAC ACCAG GAGCA-3′; reverse: 5′-GGGCG AAAGA TATGT GGGAT-3′; GAPDH forward; 5′-ACCACCTGGAGAAGGCTGG-3′: and reverse: 5′-CTCAGTGTAGCCCAGGATGC-3.

### Primary cortical neuron culture and transfection

The cerebral cortex was dissected from post-natal day 1 C57BL/6_SJL mice, dissociated with with 0.25% trypsin and plated onto 6-well plates coated with 1 mg/ml poly-L-lysine. The neurons were grown in Neurobasal medium (Invitrogen) supplemented with N2 (Invitrogen) and 100 µg/ml penicillin/streptomycin (Invitrogen) at 37 °C in a humidified environment of 95% O_2_/5% CO_2_ for DIV 7. The cells were transiently transfected with 8 µg of neuritin subcloned into a flag-IRES-EGFP vector, which was a generous gift from Dr. Nedivi at Massachusetts Institute of Technology, using a calcium phosphate method. At 48 h post-transfection, the efficiencies of the transient transfections of plasmids were confirmed via the expression of GFP fluorescence.

### Western blotting

Approximately 20–100 µg of protein was separated on a SDS/PAGE gel and transferred onto a nitrocellulose membrane (Amersham Pharmacia, Buckinghamshire, UK). The protein blot was probed with anti-synaptophysin and detected using a horseradish peroxidase-conjugated secondary antibody (GE Healthcare AB, Sweden). Immunoreactive bands were visualized using an enhanced chemiluminescence system (ECL plus: GE Healthcare).

### Primary hippocampal neuron culture and neuritin treatment

Mouse primary hippocampal neuron cultures were prepared from the hippocampi of E18 pregnant wt and Tg2576 by dissociation with 0.25% trypsin and plating onto 18 mm Φ coverslips (Marienfeld, Lauda-Konigshofen, Germany) or 6-well coated with 1 mg/ml poly-L-lysine. The neurons were grown in Neurobasal medium (Invitrogen) supplemented with B27 (Invitrogen), 0.5 mM L-glutamine (Invitrogen), 100 µg/ml penicillin/streptomycin (Invitrogen), and 2.5 µM cytosine β-D-arabinofuranoside at 37°C in a humidified environment of 95% O_2_/5% CO_2_. To evaluate dendritic spine density and morphology, neurons were plated at a density of 3×10^4^ cell/mm^2^. Neurons were treated with 150 ng/ml neuritin peptide (Abcam) at DIV 16. To measure spines and assess the maturation of individual spines, a rabies virus encoding EGFP was infected at DIV 14. At DIV 19, neurons were fixed with 4% paraformaldehyde and the dendritic spine density and the morphological changes were assessed.

### Image acquisition and quantitative morphometry

Images of rabies-EGFP virus-infected neurons were collected with an Olympus Fluoview 1000 confocal microscope using 488 line of an argon laser, the HeNe Green 543 laser, and the HeCd 442 laser. To assess the maturation stages of individual spines, images were acquired in the z dimension at optical slice thickness of 0.4 µm to cover entire neurons, using a 60×1.35 NA Plan APO oil immersion objective. Each spine was analyzed with Image J software (NIH, Bethesda, USA). Morphometric measurements and classification of dendritic spines were performed as described [17].

### BrdU staining

BrdU (50 mg/kg/day, Sigma, St. Louis, MO, USA) was injected intraperitoneally for 6 days into SD rats or wt and Tg2576 mouse with lentiviral-mediated expression of EGFP or neuritin in the IRES-EGFP lentiviral vector. Hippocampal regions were dissected from the brains isolated from the animals and processed for immunohistochemistry.

### Immunohistochemistry

Human brain tissues embedded in paraffin were cut into 4 µm-thick coronal sections and deparaffinized by oven heating and immersed in xylene for immunohistochemistry. After dehydration through a graded alcohol series to water, the slides were washed with 0.1 M Tris-buffered saline (TBS, pH.7.8) and the antigen retrieval was performed in citric acid buffer (0.01 M, pH.6.0) for 10 min at 56°C. 1% H_2_O_2_ in methanol was applied for 30 min to remove the endogenous peroxide in the tissue. Then appropriate primary antibodies were applied and incubated at room temperature overnight at 4°C and revealed by incubating the tissues with fluorescence-conjugated secondary antibodies for 1 h at room temperature (Molecular Probes, CA, USA). After 3 washes with permeabilization buffer and 1 wash with TBS, slices were mounted on microscope slides in mounting medium (DAKO, CA, USA). Frozen lentivirus-injected wt mice, Tg2576 mice or SD rat brain blocks were sectioned 30 μ and placed into a vial for free floating immunohistochemistry. 1% H2O2 in methanol was applied for 30min to remove the endogenous peroxide in the tissue. The samples were blocked with 5% bovine serum albumin in TBS with TritonX-100 and incubated with appropriate primary and secondary antibodies. Following antibody incubation, the samples were washed 3 times with TBS and placed onto a glass slide to mount. Confocal microscopic observation was performed using a Zeiss LSM510 confocal microscope (Jena, Germany).

### Statistical Analysis

The data are expressed as the means ± standard error of the mean (SEM) values. A Student's *t*-test, analysis of variance (ANOVA) or repeated measures ANOVA tests (SPSS, IL, USA) were conducted to determine statistical significance where *p*<0.05 was considered to be significant. The Bonferroni adjustment was also considered to control the level of type I error for *post hoc* multiple testings.

## Results

### Neuritin is down-regulated in the brains of AD patients

**Figure 1:**
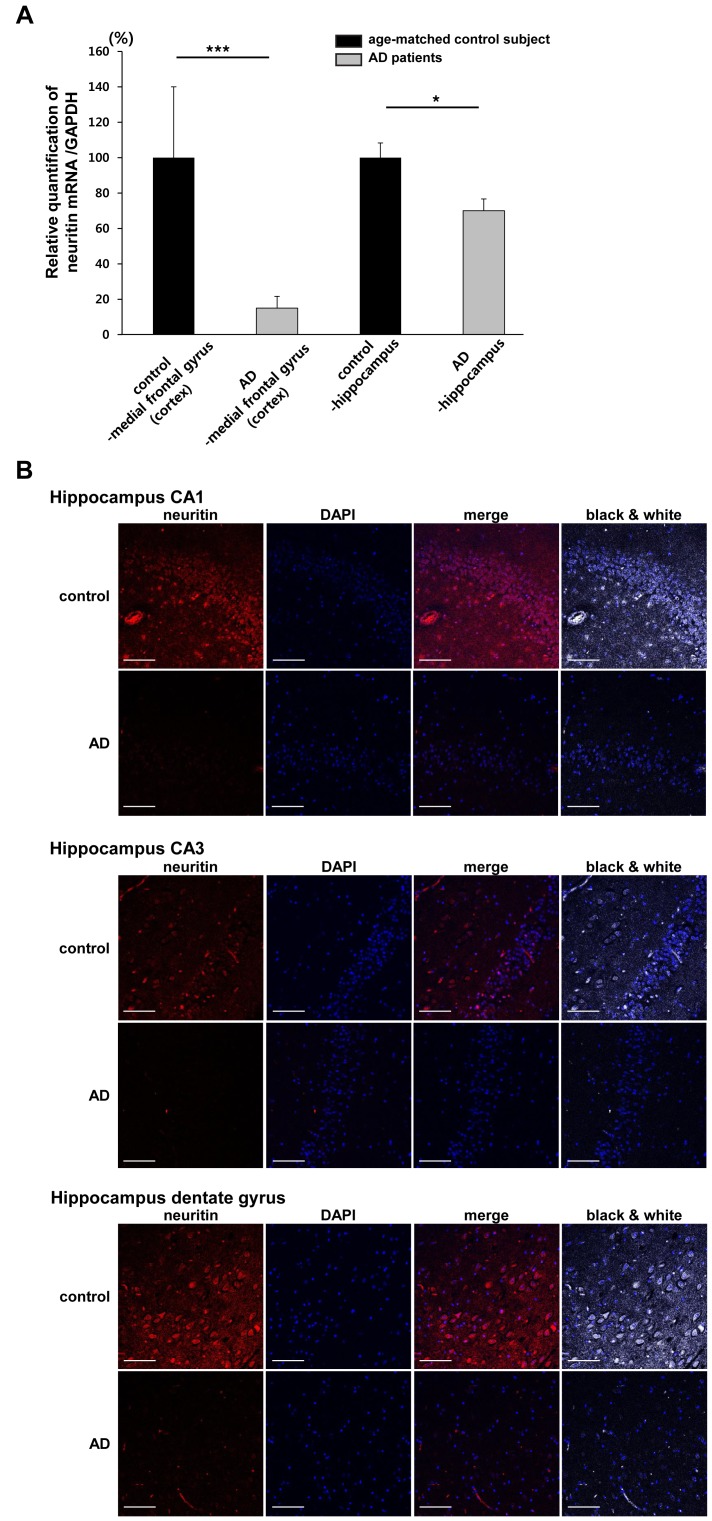
Neuritin is down-regulated in human AD brains. (A) Neuritin mRNA levels in the cortex (medial frontal gyrus) and hippocampus of age-matched control subjects (69, 79 and 80 years old) and AD patients (67, 75, 81 and 87 years old) were evaluated by qRT-PCR. Neuritin mRNA levels in the cortex and hippocampus of the AD brains were significantly lower than those of the age-matched control subjects. The relative ratio of neuritin to GAPDH mRNA in the AD patients was 15.10±6.28% compared to control subjects in cortical regions and 69.16±5.95% compared to the control subjects in the hippocampus (***p<0.001 and *p<0.05, respectively, Student's *t*-test). Data are presented as the mean ± SEM. (B) Human brain tissues embedded in paraffin were cut into 4 µm-thick coronal sections and were used to perform immunohistochemistry with an anti-neuritin antibody (red) and DAPI (blue) staining. The fluorescent images were collected from the dentate gyrus, the CA1 and CA3 regions of the hippocampus on an LSM 510 confocal microscope (Zeiss). The black and white images merged with DAPI staining are also shown. The results are representative of 3 human AD brains and 3 age-matched control brains. Scale bars represent 100 µm.

First, we examined the levels of neuritin mRNA in the medial frontal gyrus (cerebral cortex) and hippocampus of AD brains and age-matched control subjects by performing qRT-PCR. Neuritin mRNA levels in the cortex and hippocampus of AD brains (67, 75, 81 and 87 years old) were significantly lower than those of the age-matched control subjects (69, 79 and 80 years old). The relative ratio of neuritin to GAPDH mRNA in the AD patients was 15.10±6.28% compared to the control subjects in the cortical regions and 69.16±5.95% compared to the control subjects in the hippocampus (*p*<0.001 and *p*<0.05, respectively, by a Student's *t*-test, Fig. 1A). Neuritin immunoreactivity in the dentate gyrus, the CA1 and CA3 regions of the AD brains was also considerably decreased compared to the same regions of the age-matched control brains as determined by immunohistochemical methods (Fig. 1B).

### Neuritin attenuates learning and memory impairments in Tg2576 mice

**Figure 2:**
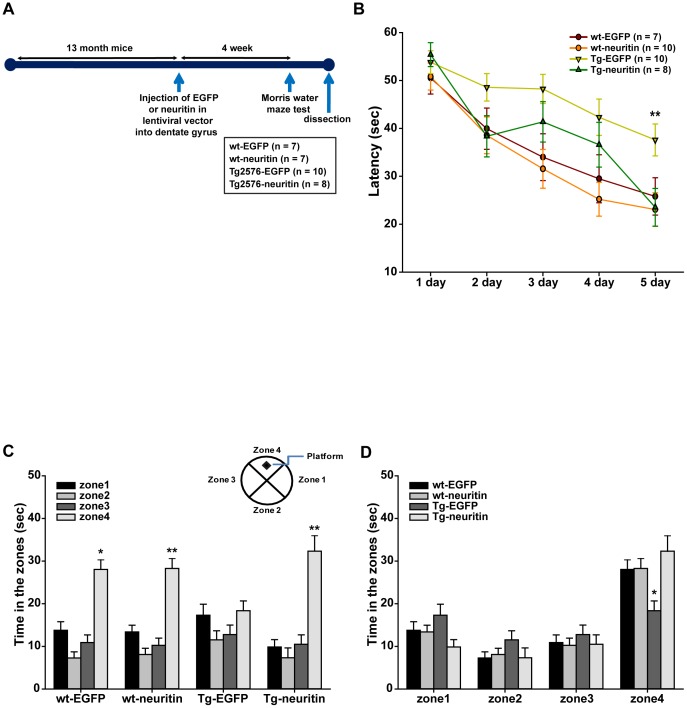
Lentiviral-mediated expression of neuritin attenuates learning and memory impairments in Tg2576 mice. (A) A schematic of the experimental scheme is shown. Viral particles of EGFP or neuritin lentiviral vector were injected into the dentate gyrus of 13-month-old wt or Tg2576 mice using a stereotaxic apparatus. (B) The Morris water maze training test was performed over 5 days. 4 weeks after the lentiviral-mediated expression of EGFP or neuritin, we tested the wt-EGFP (n = 7), wt-neuritin (n = 10), Tg2576-EGFP (n = 10) or Tg2576-neuritin (n = 8) injected mice for spatial learning and memory capability using the Morris water maze test. The 13-month-old Tg2576 mice with EGFP expression showed longer latency times than the remaining 3 groups of mice including the age-matched Tg2576 mice with neuritin expression at the fifth training days (**p<0.01, repeated measures ANOVA). (C) (D) To confirm whether the memory impairments observed in the Tg2576 mice were attenuated by neuritin expression, a probe test was performed 24 h after the 5^th^ training day and the average latency without platform in zone 4, where the platform was placed during the training period, was recorded. The wt-EGFP, wt-neuritin and Tg2576-neuritin mice but not the Tg-EGFP mice spent significantly more time in zone 4 than in the other zones (zones 1, 2 and 3) (**p<0.01, two-way ANOVA followed by *post hoc* Bonferroni test).

Next, we tested whether lentiviral-mediated expression of neuritin ameliorates the learning and memory deficits observed in Tg2576 mice, a transgenic AD animal model. We injected an EGFP or neuritin in IRES-EGFP lentiviral vector into the dentate gyrus of 13-month-old wt or Tg2576 mice using a stereotaxic apparatus. A schematic diagram of the experimental procedure is shown in Fig. 2A. First, we investigated whether GFP fluorescence was expressed in the dentate gyrus of the brains from mice with lentiviral EGFP expression 2 weeks after injection and found that GFP fluorescence was expressed in the dentate gyrus of the animals (Fig. S1). In addition, we analyzed which cell type was infected with the EGFP lentiviruses in the dentate gyrus by performing immunohistochemistry with NeuN and GFAP antibodies. We found that GFP fluorescence was almost equally observed in both NeuN-stained cells and GFAP-stained cells (Fig. S2), indicating that lentiviral particles were able to equivalently infect both neurons and astrocytes.

4 weeks after injection, we tested wt-EGFP (n = 7), wt-neuritin (n = 10), Tg2576-EGFP (n = 10) or Tg2576-neuritin (n = 8) mice for spatial learning and memory capability using a Morris water maze test. The Tg2576 mice with EGFP expression showed longer latency times than the remaining 3 groups of mice, including the age-matched Tg2576 mice with neuritin expression (Fig. 2B). The plots demonstrated a decreasing pattern except for the Tg2576-EGFP mice, in which the overall curve was located above the others. Repeated measures ANOVA indicated a statistically significant time trend across the time-points, in addition to a significant difference in mean latency time between the groups (*p*<0.01 for both, with F(4, 404) = 30.090 and F(3, 101) = 7.718, respectively). There was no significant interaction between time and group (*p* =  not significant (*n.s.*) with F(12, 404)  = 1.056, Fig. 2B).

We also performed a probe test 24 h after the 5^th^ training day and recorded the average latency without platform in zone 4, where the platform was placed during the training period. The wt-EGFP, wt-neuritin and Tg2576-neuritin mice spent significantly more time in zone 4 than in the other zones (zones 1, 2 and 3). A two-way ANOVA with the zone and the group as factors showed a significant difference in the time spent in the zone (*p*<0.01, F(3, 224) = 59.653) and a significant interaction between the zone and the group (*p*<0.01, F(9, 224) = 3.831). As observed in Fig. 2C and 2D, the wt-EGFP, wt-neuritin and Tg2576-neuritin mice but not the Tg-EGFP mice spent more time in zone 4. *Post hoc* comparisons were conducted, examining the differences between the groups in the time spent in each zone, and the null hypothesis of equal time spent by all groups was rejected significantly for zone 4 (*p*<0.01, F(3, 56) = 5.355), with a significance level of 0.0125 according to the Bonferroni adjustment for multiple tests.

**Figure 3:**
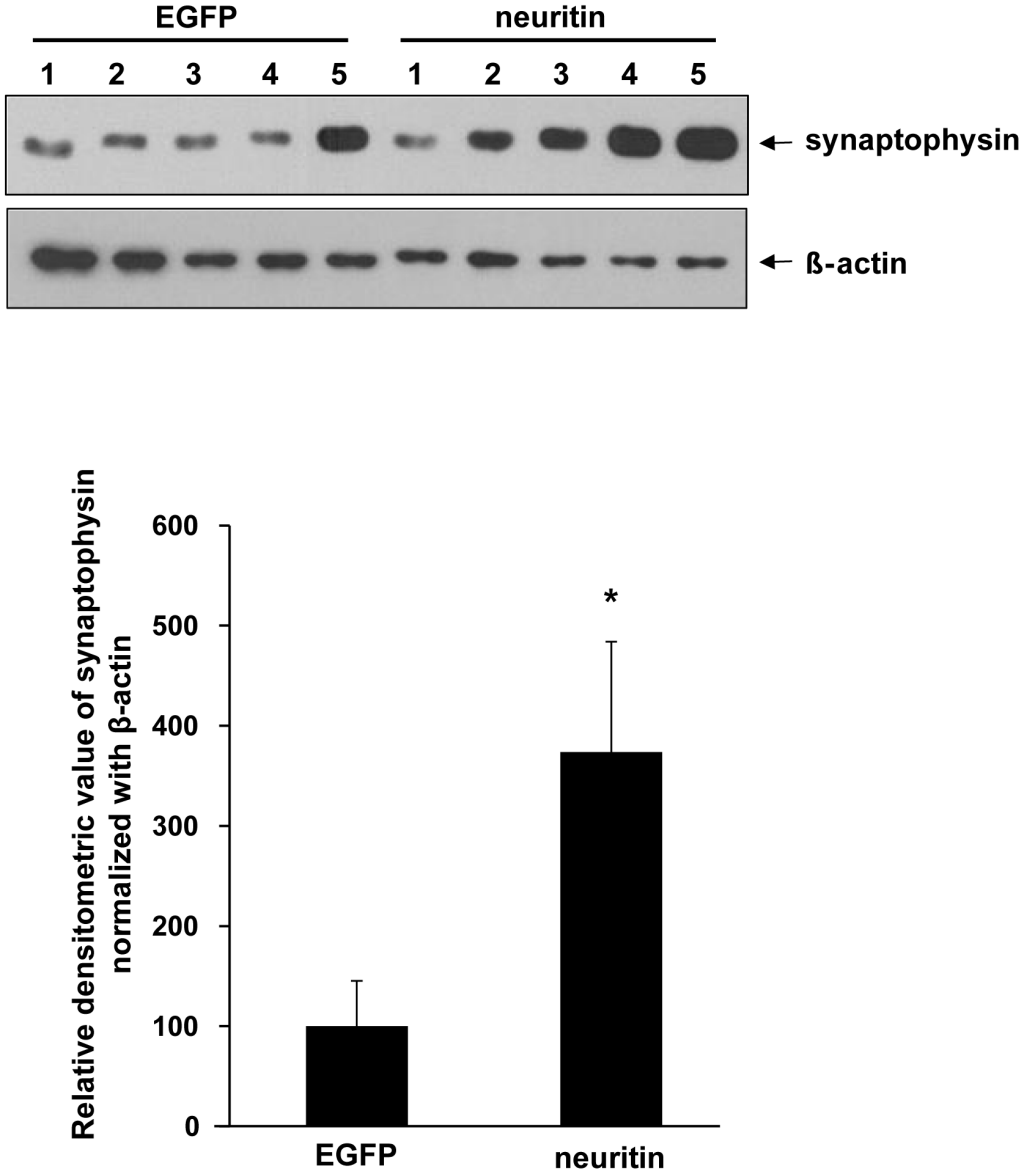
Neuritin increases synaptophysin expression in rat primary neuron cultures. Primary cultured cortical neurons were transfected at DIV 10 with EGFP or neuritin subcloned in a flag-IRES-EGFP vector by employing a calcium phosphate method. 3 days after transfection, the cells were lysed and Western blotting analysis was performed with an anti-synaptophysin antibody. β-actin was used as a loading control. Densitometric analysis was also performed. Neuritin transfection significantly increased synaptophysin expression levels by approximately 3.6-fold (*p<0.05, Student's *t*-test). The data are presented as the mean ±SEM (n = 5 dishes from 5 independent cultures for each group).

We hypothesized that the ameliorating effects of neuritin for behavioral deficits result from its synaptogenic activity. To test this, we examined synaptophysin protein levels, a presynaptic marker, in primary cortical neuron cultures (5 dishes from 5 independent cultures for EGFP or neuritin). In the neuron cultures, neuritin expression caused an approximate 3.6-fold increase in synaptophysin levels (*p*<0.05, by a Student's *t*-test, Fig. 3).

### Neuritin reverses the reduction in the dendritic spine density and the maturity of individual spines in Tg2576 neurons

**Figure 4:**
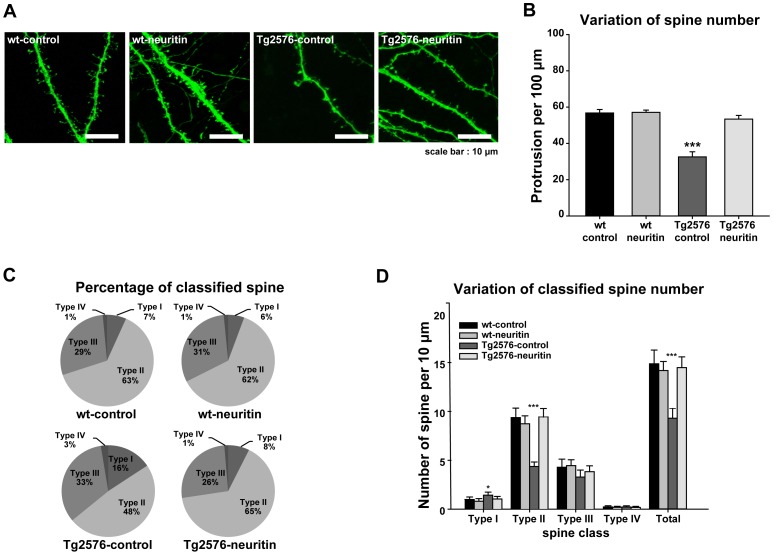
Neuritin restores the reduction in the dendritic spine density and the maturity of individual spines in Tg2576 neurons. (A) Representative images of dendrites and spines of rabies virus encoding EGFP-infected neurons are shown in the designated conditions. Scale bar represents 10 µm. (B) A summary histogram shows mean numbers of spines. Neuritin peptide (150 ng/ml) was added as a treatment for 3 days, and the dendritic spine density was then analyzed. The reduction in dendritic spine density in Tg2576 neurons (32.57±2.80/100 µm) was significantly increased to match the normal control levels in wt neurons (56.75±1.95/100 µm). (C) Proportion of spines (type I, II, III and IV) were analyzed by their morphology. Tg2576 neurons revealed a significant decrease particularly in type II spines (mushroom type; mature spines with a large head, >0.6 µm, and a short neck), but yielded a slightly higher density of type I spines (stubby type; less mature spines, relatively small spine head, <0.6 µm, and short neck, <0.3 µm) compared to the other groups. (D) A summary histogram showing mean numbers of each type of spine in the designated conditions (*p<0.05, ***p<0.001, one-way ANOVA followed by *post hoc* Bonferroni test).

We first confirmed that the dendritic spine density in primary hippocampal neuron cultures prepared from the Tg2576 mice was significantly decreased by approximately 43% compared to that from the wt mice [*p*<0.001, F(3, 116) = 23.639, by a one-way ANOVA followed by *post hoc* Bonferroni test, wt-control (n = 11), 56.75±1.95/100 µm, Tg2576-control neurons (n = 11), 32.57±2.80/100 µm; Fig. 4A, B], which is consistent with a recent report that neurons prepared from Tg2576 mice exhibited abnormal morphologies and lower spine density compared to neurons from wt control mice [18]. Given the ability of neuritin to promote synaptogenesis, we examined whether treatment with neuritin peptide affects dendritic spine density in primary hippocampal neurons. Interestingly, the dendritic spine density in Tg2576 neurons treated with neuritin peptide 150 ng/ml for 3 days (n = 11, 53.39±6.78/100 µm) was significantly increased to match the control level of wt-control neurons (n = 11, 56.75±1.95/100 µm), whereas the same treatment with neuritin in wt-neurons (n = 11, 57.12±4.11/100 µm) had no effect on the dendritic spine density in wt control neurons (n = 11, 56.75±1.95/100 µm, *p* = n.s. Fig. 4A, B).

It is widely accepted that spine morphology varies depending on activity and the maturity of individual spines [19]. We classified the spines into type I, II, III and IV in accordance with their morphology based on the established criteria as previously described [20]. Tg2576 neurons revealed a significant decrease in type II spines (mushroom type; mature spines with a large head, >0.6 µm, and a short neck), but yielded a slightly higher density of type I spines (stubby type; less mature spines, relatively small spine head, <0.6 µm, and short neck, <0.3 µm), compared to the other groups (type I spines: n = 30, *p*<0.05, F(3, 116) = 3.647, by a one-way ANOVA followed by *post hoc* Bonferroni test comparing Tg2576-control to wt-control, wt-neuritin, and Tg2576-neuritin; type II spines: *p*<0.001, F(3, 116) = 39.306; by a one-way ANOVA followed by *post hoc* Bonferroni test comparing Tg2576-control to wt-control, wt-neuritin, and Tg2576-neuritin; Fig. 4C, D, Table S1). Neuritin peptide restored the number and proportions of each type of spine in Tg2576 neurons, especially type I and type II spines (type I spines: wt-control, 0.99±0.13 spines/10 µm, 6.68±0.88% of total spines *vs.* wt-neuritin, 0.81±0.13/10 µm, 5.59±0.90% *vs.* Tg2576-control, 1.43±0.15/10 µm, 15.66±1.62% *vs.* Tg2576-neuritin, 1.04±0.13/10 µm, 7.36±0.94%; type II spines: wt-control, 9.37±0.48/10 µm, 63.24±2.18% *vs.* wt-neuritin, 9.04±0.38/10 µm, 61.88±2.21% *vs.* Tg2576-control, 4.37±0.23/10 µm, 48.34±2.10% *vs.* Tg2576-neuritin, 9.44±2.36/10 µm, 65.23±1.87%; Fig. 4C, D, Table S1). These results indicate that neuritin can rescue the deficit of synapse maturation in Tg2576 neurons, which is supported by the recovery of the number of mature spines (type II spines) (Fig. 4D, Table S1). Thus, released neuritin peptide likely functions to maintain the number of mature spines within a normal range which at least in part contributes to the amelioration of cognitive ability in Tg2576 mice.

### Neuritin up-regulates neurogenesis in the dentate gyrus of SD rats

Recent studies indicate that neurogenesis in the mature brain plays an important role in maintaining synaptic plasticity and memory formation in the hippocampus [21]. There is controversy regarding whether neurogenesis is decreased or increased in AD patients and transgenic animal models. First, we examined whether neurogenesis was altered in 13-month-old Tg2576 mice compared to wt mice. Neurogenesis as assessed with BrdU/NeuN staining, was not significantly altered in the 13-month-old Tg2576 mice compared to the age-matched wt mice (Fig. S3). Because we could not observe a significant difference between the 13-month-old wt and Tg2576 mice and evidence of neurogenesis was difficult to detect in the aged mice, 7-week-old SD rats (which are easier to handle for stereotaxic injection than are mice) were used to evaluate the effects of neuritin on neurogenesis rather than to investigate the effects of neuritin on the neurogenesis in the context of AD.

**Figure 5:**
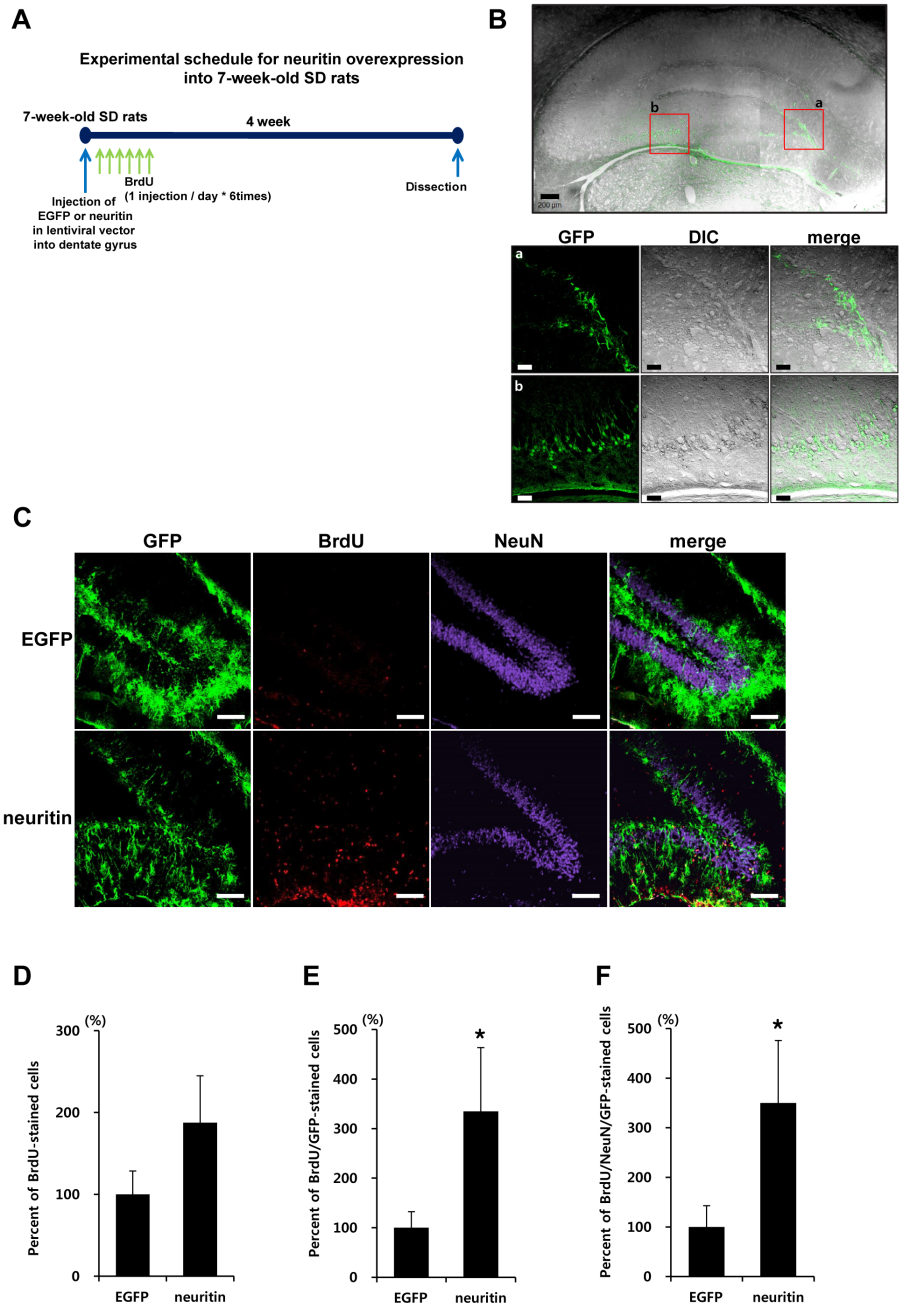
Neuritin up-regulates neurogenesis in the dentate gyrus of 7-week-old SD rats. (A) A experimental scheme is shown. 7-week-old male SD rats were injected with EGFP- or neuritin-lentiviral particles in the dentate gyrus of the hippocampus using a stereotaxic apparatus (n = 6 for each group). BrdU was intraperitoneally administered for 6 days. (B) After 2 weeks, GFP expression was confirmed by confocal laser scanning microscopy. The scale bars indicate 200 µm (low-scaled panel) and 50 µm (magnified panel). (C) Brain sections were stained with anti-GFP (green), anti-BrdU (red), and anti-NeuN (purple) antibodies. Orthogonal analysis of the staining was conducted by confocal microscopy. (D) Cells stained with BrdU antibody were counted. (E) Staining with anti-GFP and anti-BrdU antibodies was performed and counted. (F) Staining with anti-BrdU, anti-NeuN and anti-GFP antibodies was performed and counted. The results were normalized as percentage ratios to the staining in EGFP expressing rats. Rats with neuritin expression showed an increase in BrdU/GFP expressing cells by 330.77±130.78% compared to the rats with EGFP expression. Quantitative analysis of triple staining with BrdU/NeuN/GFP antibodies showed that neuritin expression induced a significant increase of newly generated neuronal cells in the dentate gyrus by 338.46±123.08% compared to EGFP-injection (*p<0.05, Student's *t*-test).

We injected EGFP or neuritin lentiviral particles into the dentate gyrus of 7-week-old male SD rats (n = 6 for EGFP and neuritin, respectively) using a stereotaxic apparatus (a schematic diagram of the experimental procedure is shown in Fig. 5A). BrdU was administered intraperitoneally once a day for 6 days (50 mg/kg/day) to label dividing cells. 2 weeks following injection, the brains were dissected. We confirmed GFP fluorescence in the dentate gyrus of EGFP or neuritin lentiviral vector- injected SD rats using confocal laser scanning microscopy (Fig. 5B).

SD rats with neuritin expression showed an increase in BrdU/GFP- expressing cells by 330.77±130.78%, compared to rats expressing EGFP (*p*<0.05, by a Student's *t*-test, Fig. 5E). Although total BrdU incorporation in the dentate gyrus of neuritin-injected SD rats was up-regulated compared to the EGFP-injected group (Fig. 5D), this increase was not statistically significant. The results in Fig. 5D and Fig. 5E are different in terms of statistical significance, which could be due to differences in the efficiency of the EGFP or neuritin infections. Quantitative analysis of triple staining with BrdU/NeuN/GFP antibodies showed that neuritin expression induced a significant increase in newly generated neuronal cells in the dentate gyrus by 338.46±123.08% compared to EGFP-injection (*p*<0.05, by a Student's *t*-test, Fig. 5F).

## Discussion

In AD, synaptic dysfunction and failure are closely related to the clinical symptoms [22]. In this study, we investigated a role for neuritin in the cellular and cognitive features of AD. We found that the cortical and hippocampal expression of neuritin are diminished in the brains of AD patients compared to age-matched control subjects (Fig. 1). Neuritin is a neuronal activity-regulated gene [7] that promotes synaptic maturation and axon arbor elaboration [11], [15], [23]. Axonal and dendritic arborization and the distribution of synapses determine the connectivity between neurons, their integrative properties and their function within a neural circuit [15], [24]. The elaboration of presynaptic axonal arbors and postsynaptic dendritic arbors and synaptogenesis occur simultaneously during brain development [25]. In a membrane-bound form linked by GPI, neuritin functions in a non-cell autonomous manner to coordinately regulate the growth of opposing dendritc and axonal arbors and to promote synaptogenesis [6], [26]–[28]. Concurrently, the secreted form of neuritin rescue cortical progenitor cells from apoptosis by preventing the activation of caspase 3 during early development and synaptic maturation [14]. In addition to its role in adults, during the development of the mammalian visual system, neuritin coordinates growth in presynaptic neurons and recruits functional AMPA receptors into synapses during activity-dependent synaptic rearrangement [5], [15].

The hippocampal expression of neuritin is decreased by chronic unpredictable stress, and treatment with antidepressants reverses this effect. Furthermore, viral-mediated expression of neuritin in the hippocampus produces antidepressant actions, prevents the atrophy of dendrites and spines, and inhibits depressive and anxiety behaviors caused by chronic unpredictable stress [23].

In the current study, we showed that the lentiviral-mediated expression of neuritin in the dentate gyrus attenuates the learning and memory deficits exhibited by Tg2576 mice in the Morris water maze test (Fig. 2). Furthermore, neuritin restores the dendritic spine density and maturity of individual spines observed in primary hippocampal neuron cultures from Tg2576 mice (Fig. 4). The effects of neuritin on dendritic spine density and morphology contribute to its ameliorating action in behavioral impairments. Notably, our report is the first to show that neuritin affects the maturity of individual spines. In addition, level of synaptophysin protein was significantly increased by neuritin expression in rat primary cortical neuron cultures (Fig. 3). In AD brains, synapses in the outer molecular layer of the hippocampal dentate gyrus are significantly reduced by approximately 20% [29], [30]. By employing a stereologic sampling scheme coupled with transmission electron microscopy using short post-mortem interval biopsy samples, it has been shown that individuals with mild AD have fewer synapses (55%) than those with mild cognitive impairments or non-cognitive impairments. The volume of the stratum radiatum of the CA1 region is reduced in mild AD compared to mild cognitive impairments or non-cognitive impairments group [31].

Mammalian memory is the result of the interaction of millions of neurons in the brain and their coordinated activity. Alterations of synaptic plasticity, such as long-term potentiation, are recognized candidate mechanisms for memory. Adult neurogenesis in the dentate gyrus is a complex, multi-step process starting with the proliferation of neural precursors that reside in the dentate subgranular layer [32]. The adult-born neurons that survive the initial period of cell death during which at least 50% of the daughter cells die within a few days, mainly differentiate into granule neurons. These mature neurons are integrated into the dentate circuits, where they receive functional inputs [33], forming functional synapses with their target cells [34], [35].

Studies of AD brains [36] and Tg animal models have revealed significant alterations in the adult neurogenesis in the hippocampus [37]–[39]. Although decreased neurogenesis is generally thought to be responsible for learning and memory impairments in AD [37], [38], [40]–[42], it is somewhat controversial whether neurogenesis is decreased or increased in AD context, based on Tg animal models or aged animal models. In addition, human autopsy studies aiming for hippocampal neurogenesis have proven difficult to interpret appropriately because the available human tissue normally reflects late stage AD; furthermore those data are confounded by variations in post-mortem delay, treatments administered, cause of death, age at death, and differing labeling techniques used to assess neurogenic activity [43].

We evaluated the evidence of neurogenesis in the dentate gyrus of 13-month-old Tg2576 mice and observed that neurogenesis as assessed with BrdU/NeuN staining was not significantly altered in this region, compared to wt mice (Fig. S3). Previous studies from other labs have produced incongruent data; Tg2576 mice showed decreased BrdU-positive cells in the dentate gyrus at 3, 6 and 9 months of age [37]. Meanwhile, PDGF-APP_Swe_ mice showed increased BrdU-positive cells in the dentate gyrus at 8 months of age [44]; Tg 9291 mice which harbor human APP 695 with Swedish, Dutch and London mutations under the Prp promoter showed increased proliferating cells stained with Ki67/BrdU in the hippocampus at 9-12 months of age [44]. We evaluated the effects of neuritin on neurogenesis in 7-week-old SD rats (Fig. 5), instead of employing Tg2576 mice. Importantly, this manipulation of SD rats indicated that neuritin expression up-regulated neurogenesis in the dentate gyrus of animals (Fig. 5). Previously, it has been reported that 1-2 weeks after a transient global ischemic insult in mice, neuritin expression in the hippocampus was up-regulated; further, in the dentate gyrus, the number of neuritin and BrdU-positive cells increased concurrently after the injury [45]. Although this report could not determine a direct relationship between neuritin and dentate granule cell proliferation, it is possible to speculate that neuritin up-regulation is highly related with dentate granule cell proliferation.

Collectively, we demonstrate that neuritin ameliorates cognitive functional impairments in Tg2576 mice and that neuritin restores the reduction in dendritic spine density and the maturity of individual spines in Tg2576 neurons. In addition, neuritin potentially up-regulates neurogenesis, although we could not determine whether the neuritin-mediated up-regulation of neurogenesis ameliorates cognitive functional impairments in the AD animal model. As a significant difference in neurogenesis was not observed in the 13-month-old Tg2576 mouse model in our hands, we speculate that the activity of neuritin in modulating neurogenesis may not be involved in the observed cognitive impairment and in its rescue in the Tg2576 mice. In conclusion, it is likely that in the 13-month-old Tg2576 mice, neuritin exerts its effects on behavioral improvements by its effects on the dendritic synaptic density as well as the maturity of the dendritic spines.

Although further research is warranted to precisely define the relationship between neuritin and the regulation of dendritic spine density as well as the maturation of individual spines and neurogenesis, our results provide substantial evidence that neuritin possesses therapeutic potential for AD.

## Supporting Information

Figure S1 **Images of GFP fluorescence in the dentate gyrus.** GFP fluorescence was examined in the contralateral and ipsilateral part of the dentate gyrus in brains from wt mice with lentiviral EGFP expression, 2 weeks after injection.
(TIF)

Figure S2 **GFP-positive neurons and astrocytes in the dentate gyrus.** GFP expression in the dentate gyrus was evaluated by performing immunohistochemistry with NeuN and GFAP antibodies 2 weeks after infection.
(TIF)

Figure S3 **Neurogenesis was not altered in 13-month-old Tg2576 mice, compared to the same aged wt mice.** A) A schematic experimental scheme is shown. Viral particles of EGFP lentiviral vector were injected into the dentate gyrus of 13-month-old wt or Tg2576 mice using a stereotaxic apparatus. BrdU was administered intraperitoneally once a day for 6 days (50 mg/kg/day) to label dividing cells. 6 weeks following the injection, the brains were dissected. B) Neurogenesis was assessed with BrdU/NeuN staining. C) A quantitative graph is shown. Neurogenesis was not altered in 13-month-old Tg2576 mice, compared to the age-matched wt mice (Student's *t*-test).
(TIF)

Table S1 **Classified spines at each condition.** To measure spines and assess the maturation of individual spines, rabies virus encoding EGFP was infected at DIV 14. Neurons were treated with 150 ng/ml neuritin peptide at DIV 16. At DIV 19, neurons were fixed with 4% paraformaldehyde and evaluated for morphological changes.
(DOCX)
