# Supplementary figures and images for: Neuritin Attenuates Cognitive Function Impairments in Tg2576 Mouse Model of Alzheimer's Disease
Source: PLoS One. 2014 Aug 7;9(8):e104121. doi: 10.1371/journal.pone.0104121 (PMC4125179; doi:10.1371/journal.pone.0104121)

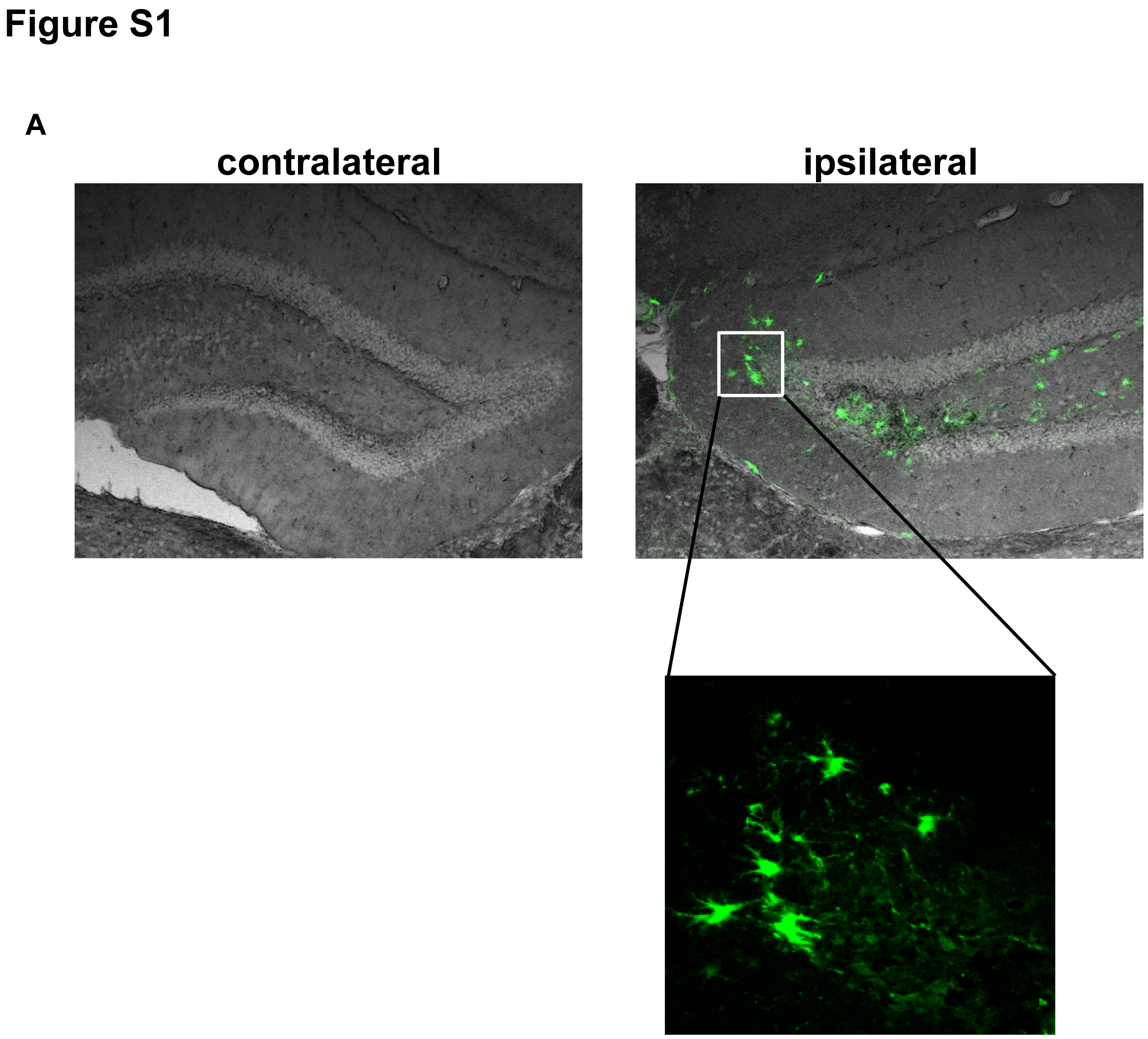

Supplement: Figure S1 — Images of GFP fluorescence in the dentate gyrus. GFP fluorescence was examined in the contralateral and ipsilateral part of the dentate gyrus in brains from wt mice with lentiviral EGFP expression, 2 weeks after injection. (TIF) [file pone.0104121.s001.tif]

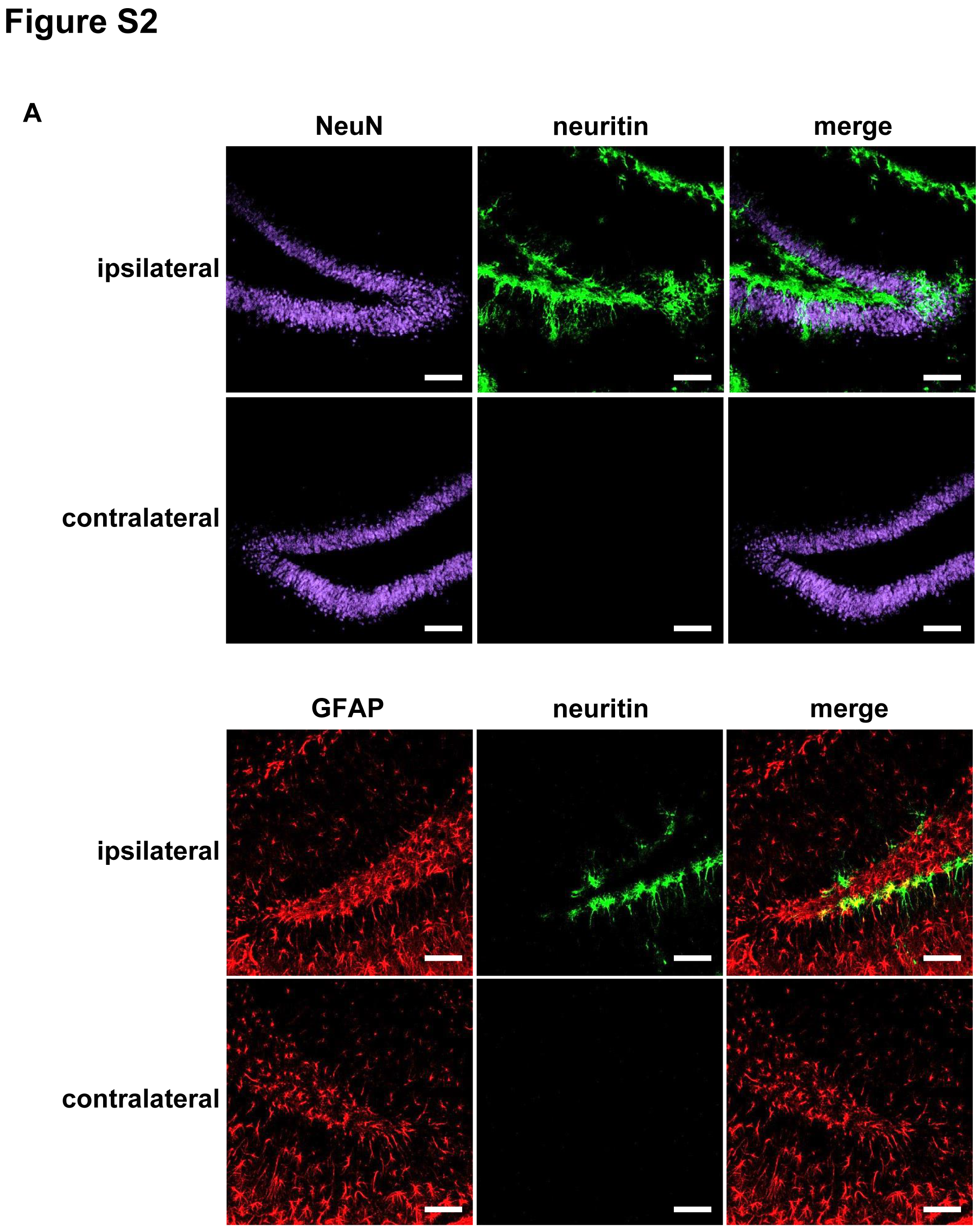

Supplement: Figure S2 — GFP-positive neurons and astrocytes in the dentate gyrus. GFP expression in the dentate gyrus was evaluated by performing immunohistochemistry with NeuN and GFAP antibodies 2 weeks after infection. (TIF) [file pone.0104121.s002.tif]

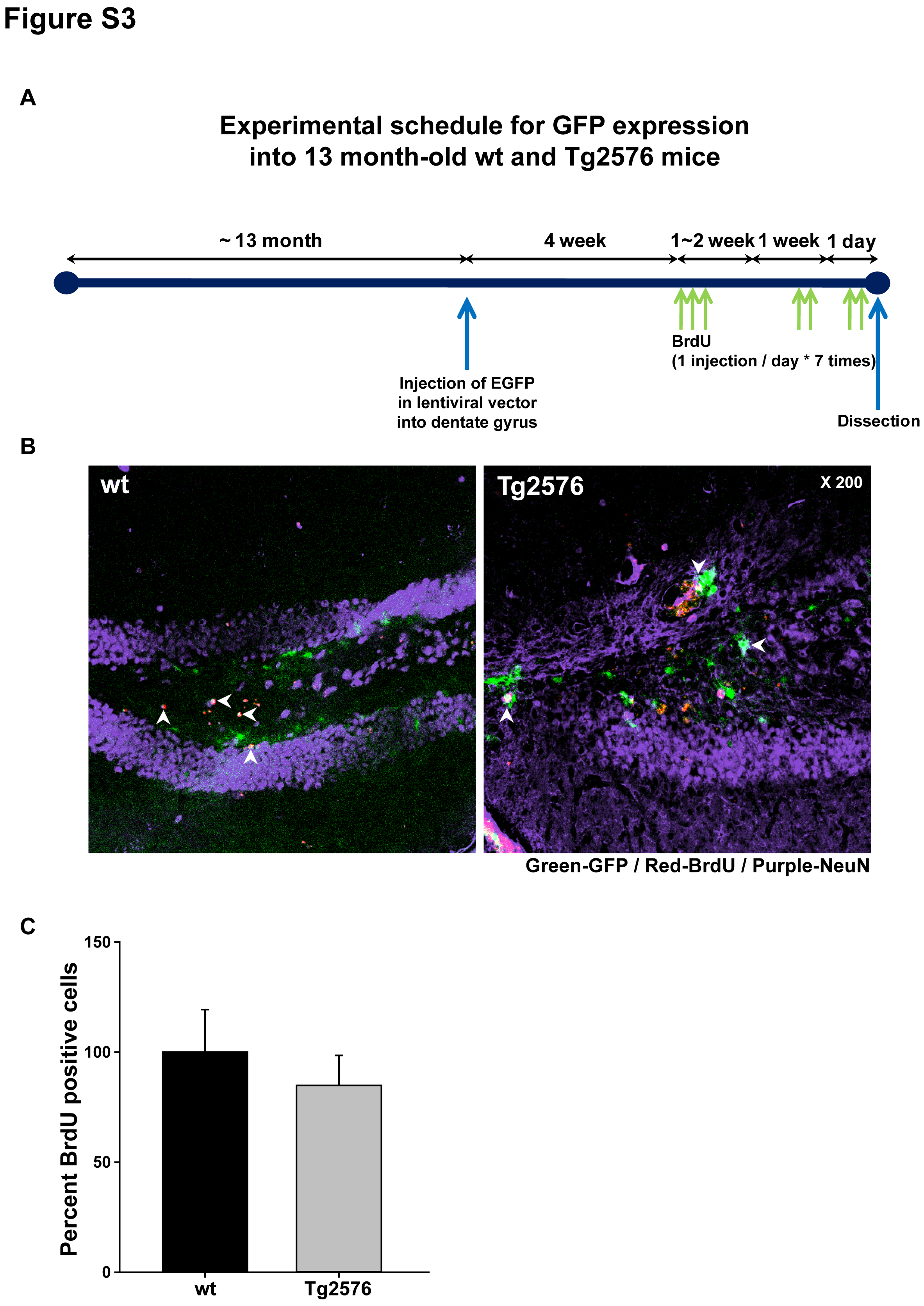

Supplement: Figure S3 — Neurogenesis was not altered in 13-month-old Tg2576 mice, compared to the same aged wt mice. A) A schematic experimental scheme is shown. Viral particles of EGFP lentiviral vector were injected into the dentate gyrus of 13-month-old wt or Tg2576 mice using a stereotaxic apparatus. BrdU was administered intraperitoneally once a day for 6 days (50 mg/kg/day) to label dividing cells. 6 weeks following the injection, the brains were dissected. B) Neurogenesis was assessed with BrdU/NeuN staining. C) A quantitative graph is shown. Neurogenesis was not altered in 13-month-old Tg2576 mice, compared to the age-matched wt mice (Student's t-test). (TIF) [file pone.0104121.s003.tif]
