# Supplementary material for: Neuritin Attenuates Cognitive Function Impairments in Tg2576 Mouse Model of Alzheimer's Disease
Source: PLoS One. 2014 Aug 7;9(8):e104121. doi: 10.1371/journal.pone.0104121 (PMC4125179; doi:10.1371/journal.pone.0104121)
Supplement: Table S1 — Classified spines at each condition. To measure spines and assess the maturation of individual spines, rabies virus encoding EGFP was infected at DIV 14. Neurons were treated with 150 ng/ml neuritin peptide at DIV 16. At DIV 19, neurons were fixed with 4% paraformaldehyde and evaluated for morphological changes. (DOCX) [file pone.0104121.s004.docx]

| **Supplementary Table 1. Descriptive of classified spines** | | | | | | | |
| --- | --- | --- | --- | --- | --- | --- | --- |
|  | | N | Mean | SD | SE | 95% Confidence Interval for Mean | |
|  |  |  |  |  |  | Lower Bound | Upper Bound |
| TypeI | wt-control | 30 | .9880 | .7107 | .1298 | .7226 | 1.2533 |
|  | wt-neuritin | 30 | .8099 | .7270 | .1327 | .5384 | 1.0813 |
|  | Tg-control | 30 | *1.430 | .8467 | .1546 | 1.1148 | 1.7471 |
|  | Tg-neuritin | 30 | 1.0413 | .7096 | .1296 | .7763 | 1.3062 |
|  |  |  |  |  |  |  |  |
| TypeII | wt-control | 30 | 9.3671 | 2.6554 | .4848 | 8.3756 | 10.3587 |
|  | wt-neuritin | 30 | 9.0365 | 2.0826 | .3802 | 8.2589 | 9.8142 |
|  | Tg-control | 30 | ***4.366 | 1.2534 | .2288 | 3.8982 | 4.8342 |
|  | Tg-neuritin | 30 | 9.4360 | 2.3582 | .4305 | 8.5555 | 10.3166 |
|  |  |  |  |  |  |  |  |
| TypeIII | wt-control | 30 | 4.2881 | 2.2664 | .4138 | 3.4418 | 5.1344 |
|  | wt-neuritin | 30 | 4.5684 | 1.6838 | .3074 | 3.9397 | 5.1972 |
|  | Tg-control | 30 | 3.2842 | 1.9543 | .3568 | 2.5545 | 4.0140 |
|  | Tg-neuritin | 30 | 3.8379 | 1.6259 | .2968 | 3.2308 | 4.4450 |
|  |  |  |  |  |  |  |  |
| TypeIV | wt-control | 30 | .2313 | .3188 | .0582 | .1122 | .3503 |
|  | wt-neuritin | 30 | .2239 | .2857 | .0522 | .1172 | .3305 |
|  | Tg-control | 30 | .2232 | .3278 | .0598 | .1008 | .3456 |
|  | Tg-neuritin | 30 | .1752 | .2986 | .0545 | .0637 | .2867 |
|  |  |  |  |  |  |  |  |
| Total | wt-control | 30 | 14.8745 | 3.8631 | .7053 | 13.4320 | 16.3170 |
|  | wt-neuritin | 30 | 14.6387 | 2.3118 | .4221 | 13.7755 | 15.5019 |
|  | Tg-control | 30 | ***9.3045 | 2.7182 | .4963 | 8.2896 | 10.3196 |
|  | Tg-neuritin | 30 | 14.4904 | 2.9958 | .5470 | 13.3718 | 15.6091 |
|  |  |  |  |  |  |  |  |
| N, dendritic segment in 11 cells for each group; SD, standard deviation; SE, standard Error | | | | | | |  |
